# Brain Activity of Benzoate, a D-Amino Acid Oxidase Inhibitor, in Patients With Mild Cognitive Impairment in a Randomized, Double-Blind, Placebo Controlled Clinical Trial

**DOI:** 10.1093/ijnp/pyab001

**Published:** 2021-01-06

**Authors:** Hsien-Yuan Lane, Cheng-Hao Tu, Wei-Che Lin, Chieh-Hsin Lin

**Affiliations:** 1 Department of Psychiatry and Brain Disease Research Center, China Medical University Hospital, Taichung, Taiwan; 2 Graduate Institute of Biomedical Sciences, China Medical University, Taichung, Taiwan; 3 Department of Psychology, College of Medical and Health Sciences, Asia University, Taichung, Taiwan; 4 Graduate Institute of Acupuncture Science, China Medical University, Taichung, Taiwan; 5 Department of Radiology, Kaohsiung Chang Gung Memorial Hospital, Chang Gung University College of Medicine, Kaohsiung, Taiwan; 6 Department of Psychiatry, Kaohsiung Chang Gung Memorial Hospital, Chang Gung University College of Medicine, Kaohsiung, Taiwan; 7 School of Medicine, Chang Gung University, Taoyuan, Taiwan

**Keywords:** Mild cognitive impairment (MCI), sodium benzoate, D-amino acid oxidase inhibitor, fMRI, regional homogeneity (ReHo)

## Abstract

**Background:**

Current anti-dementia drugs cannot benefit mild cognitive impairment (MCI). Sodium benzoate (a D-amino acid oxidase [DAO] inhibitor) has been found to improve the cognitive function of patients with early-phase Alzheimer’s disease (mild Alzheimer’s disease or MCI). However, its effect on brain function remains unknown. This study aimed to evaluate the influence of benzoate on functional magnetic resonance imaging in patients with amnestic MCI.

**Methods:**

This was a 24-week, randomized, double-blind, placebo-controlled trial that enrolled 21 patients with amnestic MCI and allocated them randomly to either of 2 treatment groups: (1) benzoate group (250–1500 mg/d), or (2) placebo group. We assessed the patients’ working memory, verbal learning and memory, and resting-state functional magnetic resonance imaging and regional homogeneity (ReHo) maps at baseline and endpoint.

**Results:**

Resting-state ReHo decreased in right orbitofrontal cortex after benzoate treatment but did not change after placebo. Moreover, after benzoate treatment, the change in working memory was positively correlated with the change in ReHo in right precentral gyrus and right middle occipital gyrus; and the change in verbal learning and memory was positively correlated with the change in ReHo in left precuneus. In contrast, after placebo treatment, the change in working memory or in verbal learning and memory was not correlated with the change in ReHo in any brain region.

**Conclusion:**

The current study is the first to our knowledge to demonstrate that a DAO inhibitor, sodium benzoate herein, can alter brain activity as well as cognitive functions in individuals with MCI. The preliminary finding lends supports for DAO inhibition as a novel approach for early dementing processes.

**Significance Statement**

This is the first study to our knowledge exploring the effect of an N-methyl-D-aspartate receptor (NMDAR) enhancer (sodium benzoate) on brain activity in the individuals with mild cognitive impairment (MCI). The benzoate treatment decreased regional homogeneity (ReHo) in the right orbitofrontal cortex, while placebo treatment did not alter ReHo. These results may indicate the possible brain mechanisms for the treatment of benzoate on MCI patients. The results may contribute to the elucidation of the pathophysiology of MCI and the development of novel therapy to enhance NMDAR.

## Introduction

Mild cognitive impairment (MCI) is a slight cognitive impairment that is accompanied by mostly normal function in processes that control the performance of daily activities (Levey et al., 2006). The concept of MCI was developed in an attempt to recognize dementia in its earliest clinically expressed form (Bowen et al., 1997; Boeve, 2012). MCI, particularly amnestic MCI (aMCI), is a risk factor or a prodromal stage of Alzheimer’s disease (AD).

Acetylcholinesterase inhibitors (AChEIs) have been developed for treating AD, especially mild-moderate AD (Birks, 2006; Burns et al., 2006). However, AChEIs are not recommended for the treatment of MCI because of the weak beneficial effects (Fellgiebel, 2007). Memantine, an N-methyl-D-aspartate (NMDA) receptor (NMDAR) antagonist, has been used to treat moderate-severe AD based on the “glutamate excitotoxicity theory” (Reisberg et al., 2003; Scarpini et al., 2003) but not for mild AD (Schneider et al., 2011). Moreover, MCI is not effectively treated by memantine either (O’Brien et al., 2011). The poor efficacy of AChEIs and memantine for MCI implies that there should be other mechanism(s) underlying the pathogenesis of MCI.

NMDAR, a subtype of ionotropic glutamate receptor, plays an important role in synaptic plasticity, learning, memory, and cognition (Wu et al., 2007; Amano and Maruyama, 2011). The NMDAR density decreases with age (Segovia et al., 2001). In AD patients, glutamate levels declined in cerebrospinal fluid (Martinez et al., 1993) and brain (Lowe et al., 1990), the number of glutamate terminals decreased in the hippocampus (Cowburn et al., 1988), and D-serine (an NMDAR agonist) levels decreased in the serum (Hashimoto et al., 2004). Therefore, dysfunction in the NMDAR neurotransmission may contribute substantially to the pathophysiology of AD.

The classical way for activation of NMDAR is applying agonists, such as D-serine (Esposito et al., 2012) or D-cycloserine (Pitkanen et al., 1995a, 1995b). However, D-cycloserine has failed in the treatment of patients with AD (Laake and Oeksengaard, 2002). A novel way to activate NMDAR is inhibiting the activity of D-amino acid oxidase (DAO) (Lin et al., 2012), which is responsible for degrading D-serine (Vanoni et al., 1997; Sasabe et al., 2012). DAO levels have been found to play a role in early-phase AD (Lin et al., 2017). Sodium benzoate, a pivotal DAO inhibitor, is generally recognized as safe and widely used as food preservatives in many countries (Joint FAO/WHO Expert Committee on Food Additives, 1973). Sodium benzoate is also used for the treatment of urea cycle enzymopathies, with the therapeutic dose in the range of 250–500 mg/kg body weight (15 000–30 000 mg for a 60-kg patient) per day (Tremblay and Qureshi, 1993; Feillet and Leonard, 1998). In a 24-week, randomized, double-blind, placebo-controlled clinical trial (Lin et al., 2014) on aMCI or mild AD, benzoate significantly improved cognitive functions (including working memory and verbal learning and memory tests). The elderly patients tolerated sodium benzoate 250–1500 mg/d very well.

Resting-state functional magnetic resonance imaging (rfMRI) is a non-invasive way to investigate the spontaneous brain activity that may reflect the brain dynamics in local cortical tissues or large-scale brain networks (Jiang and Zuo, 2016). A recent study suggested that the changes of local functional connectivity (FC) may affect whole-brain dynamics via the change of the local excitation-inhibition ratio (Deco et al., 2014). With the treatment of NMDAR antagonist MK801, increased brain responses to olfactory stimuli have been observed in primates using pharmacological fMRI (Zhao et al., 2018). The increased brain responses may be underpinned by the change of the local excitation-inhibition ratio with blockade of NMDAR on local inhibitory interneurons and then disinhibit principal neurons. Moreover, long-term exposure of ketamine, another NMDAR antagonist, has been associated the altered local FC in human (Liao et al., 2012). Hence, with treatment of sodium benzoate, the local excitation-inhibition ratio may be changed in terms of the changes in local FC. Regional homogeneity (ReHo) is a reliable method to characterized the local FC (Zang et al., 2004). In the present study, we conducted the rfMRI scans and ReHo analysis to investigate the benzoate treatment-associated local FC changes, which may contribute to the improvement of cognitive function in early-phase AD.

## Methods

This study enrolled patients with aMCI from the outpatient clinic at the Department of Psychiatry, Kaohsiung Chang Gung Memorial Hospital, Kaohsiung in a 24-week clinical trial. The institutional review board of the hospital approved the study in accordance with the current revision of the Declaration of Helsinki.

The trial was registered on the ClinicalTrials.gov website (NCTNCT02239003): https://clinicaltrials.gov/ct2/show/NCT02239003.

### Patients

After a description of the study to the patients, written informed consent was obtained. Patients were evaluated by the research psychiatrist after a thorough medical and neurological workup.

Patients were enrolled in this study if they were aged 50–90; (2) satisfied the criteria for aMCI (McKhann et al., 1984) of a presumably degenerative nature defined as subjective memory complaint corroborated by an informant and insufficient global cognitive and functional impairment to meet NINCDS-ADRDA criteria, and had a Clinical Dementia Rating (Morris, 1993) score of 0.5; (3) were physically healthy and had all laboratory assessments (including urine/blood routine, biochemical tests, and electrocardiograph) within normal limits; and (4) had sufficient education to communicate effectively and were capable of completing the assessments of the study. For patients who had already been on AChEIs therapy, AChEIs had to be continued for at least 3 months before enrollment and AChEIs doses had to be kept unchanged during the study duration. For patients who had not yet been on AChEI therapy, AChEIs or other anti-dementia medications were forbidden during the study duration.

Exclusion criteria included history of significant cerebrovascular disease; Hachinski Ischemic Score >4; major neurological, psychiatric, or medical conditions other than MCI; substance (including alcohol) abuse or dependence; delusion, hallucination, or delirium symptoms; severe visual or hearing loss; and inability to follow the protocol.

### Treatments

Eligible patients continued their originally ongoing psychotropic drugs (if any) throughout the study period and were randomly assigned to either of 2 treatment groups in a double-blind manner: sodium benzoate (250–1500 mg/d) or placebo for 24 weeks. Sodium benzoate (250 mg/capsule) was purchased from Excelsior Biopharma Inc.

To ensure concealment of the randomization assignment, study medication was provided in coded containers with supply of identical-appearing capsules of placebo or benzoate. Patients were randomized through a computer-generated randomization table to receive placebo or benzoate treatment in a 1:1 ratio. Non-blinded pharmacists dispensed appropriate medication for treatment according to the randomization table. Benzoate was initiated at 250–500 mg/d. According to the clinical condition, cognitive function assessment, and the patients’ tolerance, the dose was adjusted every 8 weeks in each group. If cognition did not improve, the dose could be titrated by 250–500 mg/d from the ninth week and another 250–500 mg/d from the 17th week of the study. The total dose range was 250–1500 mg/d. The dosing strategy of benzoate was based on the doses in our aforementioned pilot study (Lin et al., 2014), where benzoate was effective and safe after 24 weeks of treatment in the elderly patients.

During the study period, limited use of benzodiazepines (up to 4 mg/d lorazepam or equivalent) was allowed as concomitant medication for anxiety or insomnia. No other centrally acting drugs or cytochrome P450 inducers (or inhibitors) were permitted (Lane and Chang, 1998).

Patients, caregivers, and investigators, except the investigational pharmacist, were all blinded to the assignment. Patient medical adherence and safety were closely monitored by caregivers and research physicians, and pill-counting was monitored by the study staff.

### Evaluation of Cognitive Function and Side Effects

Cognitive functions were measured by working memory (Wechsler Memory Scale–Third Edition [WMS-III], Spatial Span) (Wechsler, 1997; Silver et al., 2003) and verbal learning and memory tests (WMS-III, Word Listing) (Wechsler, 1997) at week 0 and week 24, and the Alzheimer’s disease assessment scale-cognitive subscale (ADAS-cog) (Rosen et al., 1984) at weeks 0, 8, 16, and 24.

Clinical ratings were performed by a research psychiatrist (C.H.L.), who was trained and experienced in the rating scales.

Side effect assessments were examined every 8 weeks during the drug treatment period by routine physical and neurological examinations and the Udvalg for Kliniske Undersogelser Side-effects Rating Scale (Lingjaerde et al., 1987). Routine laboratory tests, including CBC and biochemistry, were checked at baseline (week 0), week 8, week 16, and endpoint of the drug treatment (week 24).

### Methods for Image Study

#### Image Acquisition

Resting-state fMRI was measured at baseline (week 0) and endpoint (week 24). Images were acquired with an 8-channel head coil in a 3.0 Tesla MRI scanner (Signa Excite, GE). The rfMRI sessions were continuously scanned with ascending echo-planar imaging sequence for whole-brain scanning (repetition time = 2000 ms; echo time = 30 ms; flip angle = 80°; matrix= 64 × 64; field of view = 240 × 240 mm^2^; slice number = 32; slice thickness = 4 mm) after 10-second blank scans for stabilized the signal. All scans were acquired within a dim-light shielding room. Before scanning, patients were instructed to remain relaxed and awakened but not to move head or focus on any specific matter.

#### Preprocessing of Resting-State fMRI Data

The preprocessing protocol of rfMRI data was reported elsewhere (Wu et al., 2016). In brief, the rfMRI images (without first 10-second blank scans) were preprocessed using Data Processing Assistant for Resting-State fMRI 4.3 (DPARSF 4.3, State Key Laboratory of Cognitive Neuroscience and Learning, Beijing Normal University, China). Images were corrected for different slice acquisition times, realigned to correct the head motions occurring alone the session, normalized into Montreal Neurological Institute reference space with echo-planar image template, and resampled with the voxel size 2 × 2 × 2 mm^3^. The time-series activities in each voxel were then linearly detrended, band-pass filtered (0.01–0.08 Hz), and regressed out the confounding variables as 6 head movement parameters, global mean signal, mean signal of white matter, and mean signal of cerebral spinal fluid. The ReHo maps were generated by calculating the Kendall’s coefficient of concordance voxel by voxel on preprocessed time-series activities between a given voxel and its nearest neighbors (26 voxels). The ReHo maps were spatially smoothed using a 3D Gaussian kernel of 8 mm full-width at half-maximum and then standardized by divided with its own global mean.

### Data Analysis

Chi-square test (or Fisher’s exact test) was used to compare differences of categorical variables, and Student 2-sample *t* test (or Mann-Whitney U test if the distribution was not normal) was used for continuous variables between 2 treatment groups.

The statistical analysis of standardized ReHo maps was conducted by statistical parametric mapping 12 (SPM12, Wellcome center for Human Neuroimaging, University College, London, UK). The 2-sample *t* test and paired *t* tests were performed to probe the possible difference between different groups at baseline and different time points (i.e., before and after treatment) in each group, respectively. The correlation analysis was also conducted between change of cognitive measurements and change of ReHo maps in each group. As an exploratory study, a less stringent significant threshold (uncorrected *P* < .005 at voxel-level with the cluster size >50, corresponding to the uncorrected cluster level *P* < .080) was applied in the present study to reduce the chance of Type II error (Cremers et al., 2017).

## Results

A total of 24 patients with aMCI were enrolled into this study. Among them, 3 patients were excluded (1 alcohol use and poor medical adherence, 1 comorbid with delusional disorder, 1 diagnosed as Parkinson disease). The remaining 21 patients completed the 24-week clinical trial and brain MRI measurement.

The demographic and clinical characteristics are shown in Table 1. Patients in the sodium benzoate group appeared younger (*P* = .042) and had a lower education level (*P* = .003) than patients in the placebo group.

**Table 1.** Demographic and Clinical Characteristics of Patients With aMCI

	Treatment groups		
	Sodium benzoate (n = 9)	Placebo (n = 12)	*P* value
Demographics			
Gender, female, n (%)	7 (77.8)	5 (41.7)	.184^*a*^
Age, year, mean (SD)	66.1 (3.2)	69.2 (3.6)	.058^*b*^
Age at illness onset, y, mean (SD)	65.4 (3.6)	68.9 (3.6)	.042^*b*^
Education, y, mean (SD)	5.0 (1.8)	8.8 (2.7)	.003^*c*^
BMI, mean (SD)	22.7 (5.8)	24.0 (2.4)	.466^*b*^
No. of patients using anti-dementia drugs			
Total	0	1	1.000^*a*^
Donepezil (dose, mean ± SD)	0	1 (5.0 ± 0.0)	1.000^*a*^

Abbreviations: aMCI, amnestic mild cognitive impairment; BMI, body mass index.

^*a*^Fisher’s exact test.

^*b*^independent *t* test.

^*c*^Mann-Whitney U test if the distribution was not normal.

At week 0 (baseline), patients in the benzoate group had a higher ADAS-cog score than the placebo group (15.1 ± 3.9 vs 10.8 ± 3.6, *P* = .023, Mann-Whitney U test). At endpoint, there was no significant difference between the 2 groups in ADAS-cog (9.1 ± 3.5 vs 6.8 ± 2.7, *P* = .113, *t* test). There was no significant difference in ADAS-cog score change from baseline to endpoint between the 2 groups (6.0 ± 2.2 vs 4.0 ± 4.0, *P* = .196, *t* test).

### ReHo at Baseline and After Treatment

At baseline, the benzoate group showed higher ReHo than in the placebo group in right middle frontal gyrus (recognized as part of orbitofrontal cortex [OFC]) and bilateral medial frontal gyrus (recognized as supplementary motor area) (supplemental Table 1).

**Figure 1.**
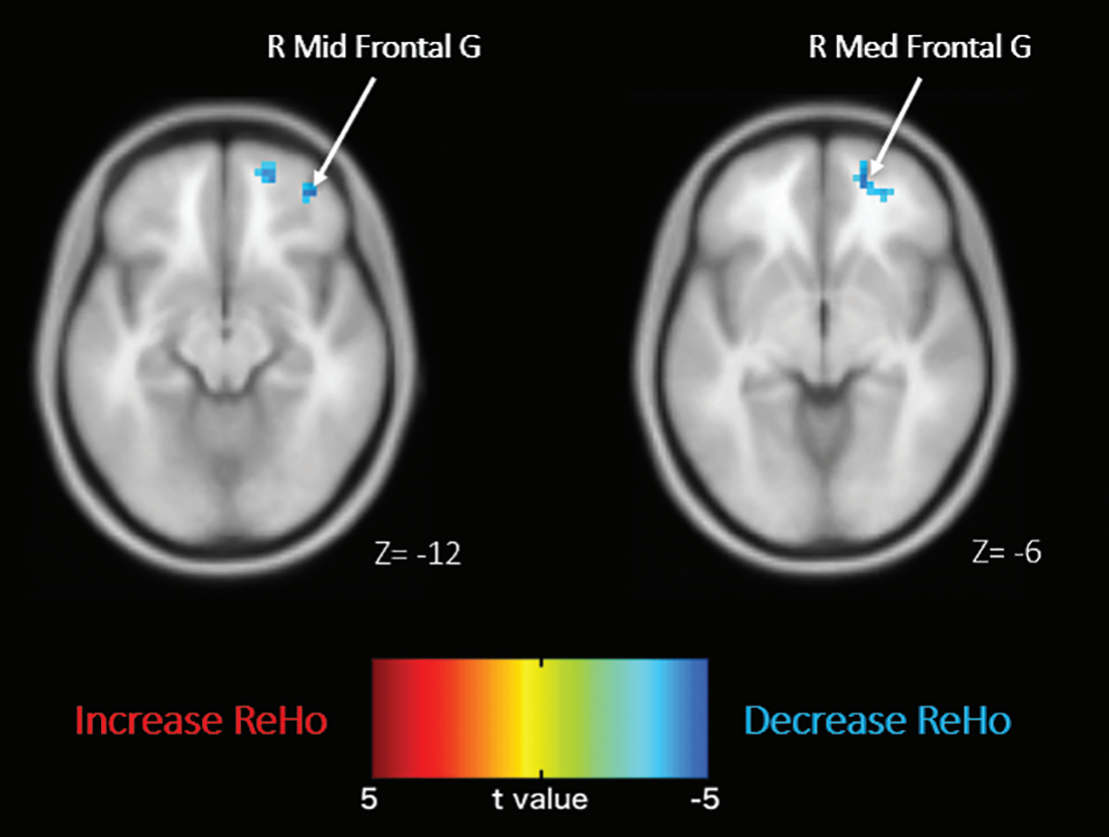
The changes of regional homogeneity after benzoate treatment in schizophrenia patients. After 24 weeks benzoate treatment, decreased regional homogeneity (ReHo) was found in right middle and medial frontal gyrus that belong to right orbitofrontal cortex. The warm and cold colors denote increased and decreased ReHo, respectively. G, gyrus; Med, medial; Mid, middle; R, right.

After benzoate treatment, decreased ReHo was found in right middle frontal gyrus, while no increased ReHo was found. After placebo treatment, no significant changes of ReHo were found (Figure 1; Table 2).

**Table 2.** Changes of ReHo After Treatment With Benzoate or Placebo

Increased ReHo							Decreased ReHo						
				Coordinates (mm)							Coordinates (mm)		
Anatomic area	BA	Size	t Score	x	y	Z	Anatomic area	BA	Size	t Score	x	y	z
Benzoate													
No significant cluster							R Mid Frontal G	11	61	4.46	39	48	−12
							R Med Frontal G	10		4.45	18	54	−6
Placebo													
No significant cluster							No significant cluster						

Abbreviations: BA, Brodmann area; G, gyrus; Med, medial; Mid, middle; R, right; ReHo, regional homogeneity; Size, number of voxels in the cluster.

### Correlations Between Cognitive Changes and ReHo Alterations

**Figure 2.**
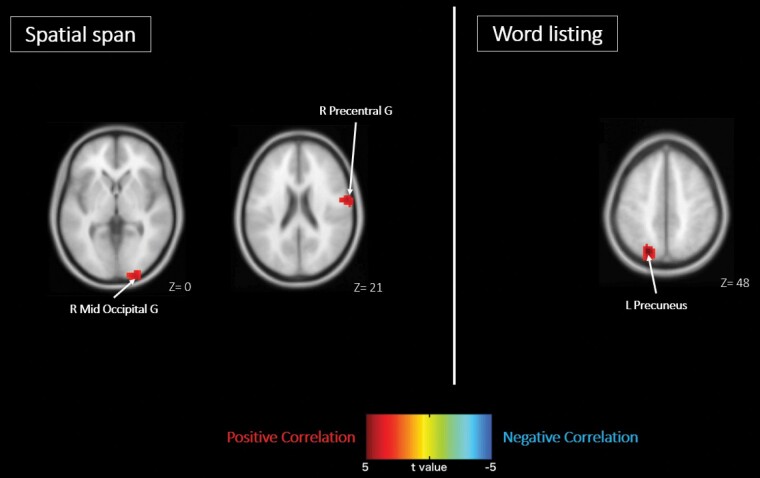
Correlation between the changes of regional homogeneity and the changes of memory tests with benzoate treatment in schizophrenia patients. The changes of regional homogeneity were positively correlated with (left) the changes of working memory in right precentral gyrus and right middle occipital gyrus and with (right) the changes of verbal learning and memory in left precuneus. The warm and cold colors denote positive and negative correlation, respectively. G, gyrus; Mid, middle; R, right.

Regarding the relationship between the change in working memory (assessed by WMS-III, Spatial Span) and the change in ReHo, a positive correlation was found in right precentral gyrus (recognized as primary motor cortex) and right middle occipital gyrus (recognized as primary visual cortex), but no negative correlation was detected in any brain region in the benzoate group. There was neither positive nor negative correlation observed in any brain region in the placebo group (Figure 2; Table 3).

**Table 3.** Correlation Between the Changes of Cognitive Tests and the Changes of ReHo With Benzoate or Placebo Treatment

Positive correlation							Negative correlation						
				Coordinate (mm)							Coordinate (mm)		
Anatomic area	BA	Size	*t* Score	x	y	z	Anatomic area	BA	Size	*t* Score	x	y	z
Wechsler Memory Scale-Third Edition, spatial span													
Benzoate													
R Precentral G	6	99	4.19	63	−3	21	No significant cluster						
R mid-occipital G	18	93	4.10	24	−102	0							
Placebo													
No significant cluster							No significant cluster						
Wechsler Memory Scale-Third Edition, word listing													
Benzoate													
L Precuneus	7	103	4.80	−21	−75	48	No Significant Cluster						
Placebo													
No significant cluster							No significant cluster						

Abbreviations: B, bilateral; BA, Brodmann area; EC, Entorhinal cortex; G, gyrus; Inf, inferior; L, left; Mid, middle; N, nucleus; PCC, posterior cingulate cortex; R, right; ReHo, regional homogeneity; Size, number of voxels in the cluster; Sup, superior.

Regarding the relationship between the change in verbal learning and memory (measured by WMS-III, Word Listing) and the change in ReHo, a positive correlation was shown in left precuneus, but no negative correlation was revealed in any brain region in the benzoate group. There was neither positive nor negative correlation discovered in any brain region in the placebo group (Figure 2; Table 3).

### Safety

Both sodium benzoate and placebo were well-tolerated. The side effect was mild and did not warrant medical treatment. All 21 patients completed the trial without dropout. The routine blood cell count and chemistry were all within the normal ranges and remained unchanged after treatment (data not shown).

## Discussion

To our knowledge, this is the first study exploring the effect of an NMDAR enhancer on brain activity in individuals with MCI or dementia. The benzoate treatment decreased ReHo in the right OFC (which consists of Brodmann area 10, 11, and 47) (Figure 1), while placebo treatment did not alter ReHo (Table 2). These results may indicate the possible brain mechanisms for the treatment of benzoate on MCI patients.

Anatomically, OFC is interconnected with parahippocampus (Haber and Behrens, 2014). It has been indicated that increased parahippocampal-prefrontal functional connectivity is predictive of impaired episodic memory in aMCI (Zhang et al., 2016). Further, the MCI patients with increased ReHo in the orbital part of the inferior frontal gyrus (which is cytoarchitectonically most closely represented by Brodmann area 47) showed a trend of deteriorating into AD or remaining in MCI rather than reverting to a cognitively intact state (Cai et al., 2018). Accordingly, in the present study, at baseline, the benzoate group also displayed greater ReHo in the right OFC and more cognitive impairment (as shown by the higher ADAS-cog score) than the placebo group.

After treatment, the mean ADAS-cog score decreased by 6.0 ± 2.2 in the benzoate group and 4.0 ± 4.0 in the placebo group; however, the group difference was insignificant in this small-sized study (*P* = .196). Of interest, as aforementioned, after benzoate treatment, decreased ReHo was found in right middle frontal gyrus, while, after placebo treatment, no significant ReHo change was found (Figure 1; Table 2). Therefore, decreased ReHo in OFC with benzoate treatment may be potentially beneficial for the outcome of the cognitive aging process. In accordance, transcranial direct current stimulation, with its potential to enhance NMDAR-related neurotransmission and neuroplasticity (Chang et al., 2018), significantly reduced prefrontal hyperactivity and resulted in “normalization” of abnormal network configuration during fMRI (Meinzer et al., 2015).

Furthermore, after benzoate treatment, the change in nonverbal (spatial) working memory was positively correlated with the change in ReHo in right precentral gyrus and right middle occipital gyrus; and the change in verbal learning and memory was positively correlated with the change in ReHo in left precuneus (Figure 2). On the other hand, after placebo treatment, the change in nonverbal working memory or in verbal learning and memory was not correlated with the change in ReHo in any brain region (Table 3). In accordance, previous study has shown that, in the nonverbal working memory, regions of activation included the precentral gyrus and precuneate gyrus in the left parietal lobe and the occipital cortex in the right hemisphere (Binder and Urbanik, 2006). In addition, in the MCI patients, perfusion in precunei, parietal cortex, and left hippocampus was correlated with verbal memory (Nobili et al., 2008).

Dysregulation of NMDAR is implicated in the pathogenesis of AD (Lin et al., 2019a, 2019b; Chang et al., 2020). DAO can regulate the NMDAR function. In a recent study in 397 individuals (including aMCI, mild AD, moderate to severe AD, and healthy elderly), DAO levels in the serum increased with the severity of the cognitive deficits (Lin et al., 2017); this is the first study indicating that the peripheral DAO levels may increase with age-related cognitive decline. The finding supports the hypofunction of NMDAR hypothesis in AD (Lin et al., 2020). It is critical to identify and treat AD as early as possible, potentially to arrest its progression (Hsu et al., 2018). Following the pilot study on sodium benzoate for the treatment of cognitive function of early-phase AD (Lin et al., 2014), the current study further supports that DAO could serve as a novel target of drug development for early stages of cognitive decline.

This study is limited by its small sample size. In addition, whether the finding in Han Taiwanese can be extrapolated to other populations is unclear.

In conclusion, the preliminary results may contribute to the elucidation of the pathophysiology of MCI and the development of novel therapy to enhance NMDAR. Future larger-sized studies in patients of various ethnicities are warranted. In addition, whether neuroimaging biomarkers, as shown in the current study, can be applied for early detection and individualized medicine also deserves further investigation.

## Supplementary Material

pyab001_suppl_Supplemental_Table_S1

pyab001_suppl_Supplementary_Materials_S1

pyab001_suppl_Supplementary_Materials_S2

## Notes

The trial was registered on the ClinicalTrials.gov website (NCTNCT02239003): https://clinicaltrials.gov/ct2/show/NCT02239003

## References

[CIT0001] Amano H , Maruyama IN (2011) Aversive olfactory learning and associative long-term memory in Caenorhabditis elegans. Learn Mem 18:654–665. 21960709 10.1101/lm.2224411 PMC3187929

[CIT0002] Binder M , Urbanik AS (2006) Material-dependent activation in prefrontal cortex: working memory for letters and texture patterns–initial observations. Radiology 238:256–263. 16304084 10.1148/radiol.2381041622

[CIT0003] Birks J (2006) Cholinesterase inhibitors for Alzheimer’s disease. Cochrane Database Syst Rev 1:CD005593. 10.1002/14651858.CD005593 PMC9006343 16437532

[CIT0004] Boeve BF (2012) Mild cognitive impairment associated with underlying Alzheimer’s disease versus Lewy body disease. Parkinsonism Relat Disord 18 Suppl 1:S41–S44. 22166451 10.1016/S1353-8020(11)70015-3

[CIT0005] Bowen J , Teri L, Kukull W, McCormick W, McCurry SM, Larson EB (1997) Progression to dementia in patients with isolated memory loss. Lancet 349:763–765. 9074575 10.1016/S0140-6736(96)08256-6

[CIT0006] Burns A et al (2006) Clinical practice with anti-dementia drugs: a consensus statement from British Association for Psychopharmacology. J Psychopharmacol 20:732–755. 17060346 10.1177/0269881106068299

[CIT0007] Cai S , Wang Y, Kang Y, Wang H, Kim H, von Deneen KM, Huang M, Jiang Y, Huang L (2018) Differentiated regional homogeneity in progressive mild cognitive impairment: a study with post hoc label. Am J Alzheimers Dis Other Demen 33: 373–384. 29847992 10.1177/1533317518778513 PMC10852514

[CIT0008] Chang CH , Lane HY, Lin CH (2018) Brain stimulation in Alzheimer’s disease. Front Psychiatry 9:201. 29910746 10.3389/fpsyt.2018.00201 PMC5992378

[CIT0009] Chang CH , Lin CH, Lane HY (2020) d-Glutamate and gut microbiota in Alzheimer’s disease. Int J Mol Sci 21:2676. 10.3390/ijms21082676 PMC7215955 32290475

[CIT0010] Cowburn R , Hardy J, Roberts P, Briggs R (1988) Regional distribution of pre- and postsynaptic glutamatergic function in Alzheimer’s disease. Brain Res 452:403–407. 2900052 10.1016/0006-8993(88)90048-0

[CIT0011] Cremers HR , Wager TD, Yarkoni T (2017) The relation between statistical power and inference in fMRI. PLoS One 12:e0184923. 29155843 10.1371/journal.pone.0184923 PMC5695788

[CIT0012] Deco G , Ponce-Alvarez A, Hagmann P, Romani GL, Mantini D, Corbetta M (2014) How local excitation-inhibition ratio impacts the whole brain dynamics. J Neurosci 34:7886–7898. 24899711 10.1523/JNEUROSCI.5068-13.2014 PMC4044249

[CIT0013] Esposito S , Pristerà A, Maresca G, Cavallaro S, Felsani A, Florenzano F, Manni L, Ciotti MT, Pollegioni L, Borsello T, Canu N (2012) Contribution of serine racemase/d-serine pathway to neuronal apoptosis. Aging Cell 11:588–598. 22507034 10.1111/j.1474-9726.2012.00822.x

[CIT0014] Feillet F , Leonard JV (1998) Alternative pathway therapy for urea cycle disorders. J Inherit Metab Dis 21 Suppl 1:101–111. 9686348 10.1023/a:1005365825875

[CIT0015] Fellgiebel A (2007) [Alzheimer drugs for mild cognitive impairment]. Neuropsychiatr 21:230–233. 17915184

[CIT0016] Haber SN , Behrens TE (2014) The neural network underlying incentive-based learning: implications for interpreting circuit disruptions in psychiatric disorders. Neuron 83:1019–1039. 25189208 10.1016/j.neuron.2014.08.031 PMC4255982

[CIT0017] Hashimoto K , Fukushima T, Shimizu E, Okada S, Komatsu N, Okamura N, Koike K, Koizumi H, Kumakiri C, Imai K, Iyo M (2004) Possible role of D-serine in the pathophysiology of Alzheimer’s disease. Prog Neuropsychopharmacol Biol Psychiatry 28:385–388. 14751437 10.1016/j.pnpbp.2003.11.009

[CIT0018] Hsu WY , Lane HY, Lin CH (2018) Medications used for cognitive enhancement in patients with schizophrenia, bipolar disorder, Alzheimer’s disease, and Parkinson’s disease. Front Psychiatry 9:91. 29670547 10.3389/fpsyt.2018.00091 PMC5893641

[CIT0019] Jiang L , Zuo XN (2016) Regional homogeneity: a multimodal, multiscale neuroimaging marker of the human connectome. Neuroscientist 22:486–505. 26170004 10.1177/1073858415595004 PMC5021216

[CIT0019a] Joint FAO/WHO Expert Committee on Food Additives (1973) Toxicological evaluation of certain food additives with a review of general principles and of specifications, seventeenth report of the Joint FAO/WHO Expert Committee on Food Additives. Geneva, Switzerland: World Health Organization. 4205977

[CIT0020] Laake K , Oeksengaard AR (2002) D-cycloserine for Alzheimer’s disease. Cochrane Database Syst Rev 2:CD003153. 10.1002/14651858.CD003153 PMC6718229 12076471

[CIT0021] Lane HY , Chang WH (1998) Risperidone-carbamazepine interactions: is cytochrome P450 3A involved? J Clin Psychiatry 59:430–431. 9721825

[CIT0022] Levey A , Lah J, Goldstein F, Steenland K, Bliwise D (2006) Mild cognitive impairment: an opportunity to identify patients at high risk for progression to Alzheimer’s disease. Clin Ther 28:991–1001. 16990077 10.1016/j.clinthera.2006.07.006

[CIT0023] Liao Y , Tang J, Fornito A, Liu T, Chen X, Chen H, Xiang X, Wang X, Hao W (2012) Alterations in regional homogeneity of resting-state brain activity in ketamine addicts. Neurosci Lett 522:36–40. 22698584 10.1016/j.neulet.2012.06.009

[CIT0024] Lin CH , Lane HY, Tsai GE (2012) Glutamate signaling in the pathophysiology and therapy of schizophrenia. Pharmacol Biochem Behav 100:665–677. 21463651 10.1016/j.pbb.2011.03.023

[CIT0025] Lin CH , Chen PK, Chang YC, Chuo LJ, Chen YS, Tsai GE, Lane HY (2014) Benzoate, a D-amino acid oxidase inhibitor, for the treatment of early-phase Alzheimer disease: a randomized, double-blind, placebo-controlled trial. Biol Psychiatry 75:678–685. 24074637 10.1016/j.biopsych.2013.08.010

[CIT0026] Lin CH , Yang HT, Chiu CC, Lane HY (2017) Blood levels of D-amino acid oxidase vs. D-amino acids in reflecting cognitive aging. Sci Rep 7:14849. 29093468 10.1038/s41598-017-13951-7 PMC5665939

[CIT0027] Lin CH , Chiu CC, Huang CH, Yang HT, Lane HY (2019) pLG72 levels increase in early phase of Alzheimer’s disease but decrease in late phase. Sci Rep 9:13221. 31520071 10.1038/s41598-019-49522-1 PMC6744481

[CIT0028] Lin CH , Yang HT, Lane HY (2019) D-glutamate, D-serine, and D-alanine differ in their roles in cognitive decline in patients with Alzheimer’s disease or mild cognitive impairment. Pharmacol Biochem Behav 185:172760. 31422081 10.1016/j.pbb.2019.172760

[CIT0029] Lin CH , Yang HT, Chen PK, Wang SH, Lane HY (2020) Precision medicine of sodium benzoate for the treatment of behavioral and psychological symptoms of dementia (BPSD). Neuropsychiatr Dis Treat 16:509–518. 32110025 10.2147/NDT.S234371 PMC7039065

[CIT0030] Lingjaerde O , Ahlfors UG, Bech P, Dencker SJ, Elgen K (1987) The UKU side effect rating scale. A new comprehensive rating scale for psychotropic drugs and a cross-sectional study of side effects in neuroleptic-treated patients. Acta Psychiatr Scand Suppl 334:1–100. 2887090 10.1111/j.1600-0447.1987.tb10566.x

[CIT0031] Lowe SL , Bowen DM, Francis PT, Neary D (1990) Ante mortem cerebral amino acid concentrations indicate selective degeneration of glutamate-enriched neurons in Alzheimer’s disease. Neuroscience 38:571–577. 1980143 10.1016/0306-4522(90)90051-5

[CIT0032] Martinez M , Frank A, Diez-Tejedor E, Hernanz A (1993) Amino acid concentrations in cerebrospinal fluid and serum in Alzheimer’s disease and vascular dementia. J Neural Transm Park Dis Dement Sect 6:1–9. 8216758 10.1007/BF02252617

[CIT0033] McKhann G , Drachman D, Folstein M, Katzman R, Price D, Stadlan EM (1984) Clinical diagnosis of Alzheimer’s disease: report of the NINCDS-ADRDA Work Group under the auspices of Department of Health and Human Services Task Force on Alzheimer’s Disease. Neurology 34:939–944. 6610841 10.1212/wnl.34.7.939

[CIT0034] Meinzer M , Lindenberg R, Phan MT, Ulm L, Volk C, Flöel A (2015) Transcranial direct current stimulation in mild cognitive impairment: behavioral effects and neural mechanisms. Alzheimers Dement 11:1032–1040. 25449530 10.1016/j.jalz.2014.07.159

[CIT0035] Morris JC (1993) The Clinical Dementia Rating (CDR): current version and scoring rules. Neurology 43:2412–2414. 10.1212/wnl.43.11.2412-a 8232972

[CIT0036] Nobili F , Frisoni GB, Portet F, Verhey F, Rodriguez G, Caroli A, Touchon J, Calvini P, Morbelli S, De Carli F, Guerra UP, Van de Pol LA, Visser PJ (2008) Brain SPECT in subtypes of mild cognitive impairment. Findings from the DESCRIPA multicenter study. J Neurol 255:1344–1353. 18958573 10.1007/s00415-008-0897-4

[CIT0037] O’Brien JT , Burns A, Group BAPDC (2011) Clinical practice with anti-dementia drugs: a revised (second) consensus statement from the British Association for Psychopharmacology. J Psychopharmacol 25:997–1019. 21088041 10.1177/0269881110387547

[CIT0038] Pitkänen M , Sirviö J, MacDonald E, Ekonsalo T, Riekkinen P Sr (1995a) The effects of d-cycloserine, a partial agonist at the glycine binding site, on spatial learning and working memory in scopolamine-treated rats. J Neural Transm Park Dis Dement Sect 9:133–144. 8526998 10.1007/BF02259655

[CIT0039] Pitkänen M , Sirviö J, MacDonald E, Niemi S, Ekonsalo T, Riekkinen P Sr (1995b) The effects of D-cycloserine and MK-801 on the performance of rats in two spatial learning and memory tasks. Eur Neuropsychopharmacol 5:457–463. 8998397

[CIT0040] Reisberg B , Doody R, Stoffler A, Schmitt F, Ferris S, Mobius HJ (2003) Memantine in moderate-to-severe Alzheimer’s disease. N Engl J Med 348:1333–1341. 12672860 10.1056/NEJMoa013128

[CIT0041] Rosen WG , Mohs RC, Davis KL (1984) A new rating scale for Alzheimer’s disease. Am J Psychiatry 141:1356–1364. 6496779 10.1176/ajp.141.11.1356

[CIT0042] Sasabe J , Miyoshi Y, Suzuki M, Mita M, Konno R, Matsuoka M, Hamase K, Aiso S (2012) D-amino acid oxidase controls motoneuron degeneration through D-serine. Proc Natl Acad Sci U S A 109:627–632. 22203986 10.1073/pnas.1114639109 PMC3258611

[CIT0043] Scarpini E , Scheltens P, Feldman H (2003) Treatment of Alzheimer’s disease: current status and new perspectives. Lancet Neurol 2:539–547. 12941576 10.1016/s1474-4422(03)00502-7

[CIT0044] Schneider LS , Dagerman KS, Higgins JP, McShane R (2011) Lack of evidence for the efficacy of memantine in mild Alzheimer disease. Arch Neurol 68:991–998. 21482915 10.1001/archneurol.2011.69

[CIT0045] Segovia G , Porras A, Del Arco A, Mora F (2001) Glutamatergic neurotransmission in aging: a critical perspective. Mech Ageing Dev 122:1–29. 11163621 10.1016/s0047-6374(00)00225-6

[CIT0046] Silver H , Feldman P, Bilker W, Gur RC (2003) Working memory deficit as a core neuropsychological dysfunction in schizophrenia. Am J Psychiatry 160:1809–1816. 14514495 10.1176/appi.ajp.160.10.1809

[CIT0047] Tremblay GC , Qureshi IA (1993) The biochemistry and toxicology of benzoic acid metabolism and its relationship to the elimination of waste nitrogen. Pharmacol Ther 60:63–90. 8127924 10.1016/0163-7258(93)90022-6

[CIT0048] Vanoni MA , Cosma A, Mazzeo D, Mattevi A, Todone F, Curti B (1997) Limited proteolysis and X-ray crystallography reveal the origin of substrate specificity and of the rate-limiting product release during oxidation of D-amino acids catalyzed by mammalian D-amino acid oxidase. Biochemistry 36:5624–5632. 9153402 10.1021/bi963023s

[CIT0049] Wechsler D (1997) Wechsler memory scale. 3rd ed. San Antonio, TX: Psychological Association.

[CIT0050] Wu CL , Xia S, Fu TF, Wang H, Chen YH, Leong D, Chiang AS, Tully T (2007) Specific requirement of NMDA receptors for long-term memory consolidation in Drosophila ellipsoid body. Nat Neurosci 10:1578–1586. 17982450 10.1038/nn2005 PMC3055246

[CIT0051] Wu TH , Tu CH, Chao HT, Li WC, Low I, Chuang CY, Yeh TC, Cheng CM, Chou CC, Chen LF, Hsieh JC (2016) Dynamic changes of functional pain connectome in women with primary dysmenorrhea. Sci Rep 6:24543. 27089970 10.1038/srep24543 PMC4835697

[CIT0052] Zang Y , Jiang T, Lu Y, He Y, Tian L (2004) Regional homogeneity approach to fMRI data analysis. Neuroimage 22:394–400. 15110032 10.1016/j.neuroimage.2003.12.030

[CIT0053] Zhang Y , Simon-Vermot L, Araque Caballero MÁ, Gesierich B, Taylor ANW, Duering M, Dichgans M, Ewers M; Alzheimer’s Disease Neuroimaging Initiative (2016) Enhanced resting-state functional connectivity between core memory-task activation peaks is associated with memory impairment in MCI. Neurobiol Aging 45:43–49. 27459924 10.1016/j.neurobiolaging.2016.04.018

[CIT0054] Zhao F , Holahan MA, Wang X, Uslaner JM, Houghton AK, Evelhoch JL, Winkelmann CT, Hines CDG (2018) fMRI study of the role of glutamate NMDA receptor in the olfactory processing in monkeys. Plos One 13:e0198395. 29870538 10.1371/journal.pone.0198395 PMC5988321

