# Supplementary material for: Brain Activity of Benzoate, a D-Amino Acid Oxidase Inhibitor, in Patients With Mild Cognitive Impairment in a Randomized, Double-Blind, Placebo Controlled Clinical Trial
Source: Int J Neuropsychopharmacol. 2021 Jan 6;24(5):392–9. doi: 10.1093/ijnp/pyab001 (PMC8130199; doi:10.1093/ijnp/pyab001)
Supplement: pyab001_suppl_Supplemental_Table_S1 [file pyab001_suppl_supplemental_table_s1.docx]

Supplemental Table S1. The changes of regional homogeneity between

benzoate and placebo before treatment

| Anatomic area | | BA | Size | t Score | Coordinates (mm) | | |
| --- | --- | --- | --- | --- | --- | --- | --- |
|  |  |  |  |  | x | y | Z |
| Benzoate > Placebo | |  |  |  |  |  |  |
|  | R Mid Frontal G | 11 | 77 | 4.61 | 39 | 48 | -12 |
|  | Bi Sup Frontal G | 6 | 96 | 4.36 | 9 | 0 | 69 |
|  |  |  |  | 3.81 | -3 | 0 | 75 |
| Benzoate < Placebo | |  |  |  |  |  |  |
|  | No significant cluster |  |  |  |  |  |  |

BA: Brodmann area; Size: number of voxels in the cluster; R: right;

Mid: middle; G: gyrus; Bi: bilateral; Sup: superior
