# Supplementary material for: Brain Activity of Benzoate, a D-Amino Acid Oxidase Inhibitor, in Patients With Mild Cognitive Impairment in a Randomized, Double-Blind, Placebo Controlled Clinical Trial
Source: Int J Neuropsychopharmacol. 2021 Jan 6;24(5):392–9. doi: 10.1093/ijnp/pyab001 (PMC8130199; doi:10.1093/ijnp/pyab001)
Supplement: pyab001_suppl_Supplementary_Materials_S2 [file pyab001_suppl_supplementary_materials_s2.doc]

**Supplementary Figure 1**. Flow diagram and disposition of the two treatment groups.

24 patients were screened

3 patients were excluded

1 Alcohol use

1 Comorbid with delusional disorder

1 Diagnosed as Parkinson disease

9 patients were given Sodium benzoate

0 patient withdrew

Efficacy and adverse effects assessed at weeks 8, 16, and 24

9 patients completed the trial and brain MRI at endpoint

21 patients were enrolled

Efficacy and adverse effects assessed at weeks 8, 16, and 24

12 patients completed the trial and brain MRI at endpoint

0 patient withdrew

12 patients were given placebo
